# Predicting the Long-Term Impact of Antiretroviral Therapy Scale-Up on Population Incidence of Tuberculosis

**DOI:** 10.1371/journal.pone.0075466

**Published:** 2013-09-17

**Authors:** Peter J. Dodd, Gwenan M. Knight, Stephen D. Lawn, Elizabeth L. Corbett, Richard G. White

**Affiliations:** 1 Centre for Mathematical Modelling of Infectious Diseases, London School of Hygiene and Tropical Medicine, London, United Kingdom; 2 TB Centre, London School of Hygiene and Tropical Medicine, London, United Kingdom; 3 Desmond Tutu HIV Centre, University of Cape Town, Cape Town South Africa; 4 HIV and TB Group, Malawi-Liverpool-Wellcome Trust Clinical Research Programme, Blantyre, Malawi; The University of Tokyo, Japan

## Abstract

**Objective:**

To investigate the impact of antiretroviral therapy (ART) on long-term population-level tuberculosis disease (TB) incidence in sub-Saharan Africa.

**Methods:**

We used a mathematical model to consider the effect of different assumptions about life expectancy and TB risk during long-term ART under alternative scenarios for trends in population HIV incidence and ART coverage.

**Results:**

All the scenarios we explored predicted that the widespread introduction of ART would initially reduce population-level TB incidence. However, many modelled scenarios projected a rebound in population-level TB incidence after around 20 years. This rebound was predicted to exceed the TB incidence present before ART scale-up if decreases in HIV incidence during the same period were not sufficiently rapid or if the protective effect of ART on TB was not sustained. Nevertheless, most scenarios predicted a reduction in the cumulative TB incidence when accompanied by a relative decline in HIV incidence of more than 10% each year.

**Conclusions:**

Despite short-term benefits of ART scale-up on population TB incidence in sub-Saharan Africa, longer-term projections raise the possibility of a rebound in TB incidence. This highlights the importance of sustaining good adherence and immunologic response to ART and, crucially, the need for effective HIV preventive interventions, including early widespread implementation of ART.

## Introduction

It is estimated that 79% of the global burden of HIV-associated tuberculosis disease (TB) occurs within sub-Saharan Africa [1]. Control of this epidemic requires not only TB case finding and effective treatment, but also preventive interventions including antiretroviral therapy (ART), isoniazid preventive therapy, intensified case finding and infection control [2]. Among these interventions, ART is the key tool, profoundly reducing all-cause mortality. ART reduces TB incidence rates in HIV cohorts over the short-term by approximately two-thirds, and this effect is observed across a broad range of baseline CD4 cell counts and independent of tuberculin skin test status [3]–[5]. Time-dependent reductions in incidence rates are strongly associated with the CD4 cell count response to ART [6], [7]. Short-term benefits in TB notification rates at the population level have also been observed in communities in South Africa and Malawi where ART has been scaled up rapidly [8], [9].

Despite these promising short-term observations, the long-term impact of ART scale-up on TB incidence rates at the population level remains unknown and will depend on a number of factors. TB rates after 8 years of follow-up in a South African ART cohort remained several-fold higher than those in non-HIV-infected people in the same community, even among those with the greatest CD4 cell count recovery [10]. Longer-term responses to ART are unknown but as ART services have expanded over time, reports from the region describe increasing rates of programme loss to follow-up and virological failure [11], [12]. Thus, individuals receiving ART are likely to retain a high cumulative lifetime risk of TB. Compounded by rising HIV prevalence resulting from increased life expectancy with ART scale-up, and ongoing HIV transmission, these factors could collectively result in a rebound in population level TB incidence rates in the long-term.

The potential for a rebound in TB incidence may be offset by decreases in HIV incidence, either due to the natural dynamics of the HIV epidemic or to preventive interventions including ART. There is now much interest in the potential for widespread scale-up of early ART (or universal test and treat) to reduce HIV incidence [13], [14]. This strategy could also reduce TB incidence due to the combined effects of short-term CD4 recovery and long-term reductions in HIV incidence [5], [15].

No empirical studies have yet measured the long-term dynamics of protection from TB in individuals on ART, nor the impact of combinations of ART and other HIV-prevention measures on population-level HIV-incidence. We therefore investigated how uncertainty in these variables is reflected in long-term population-level TB trends, using a mathematical model that separates the contributions from each.

## Methods

**Figure 1:**
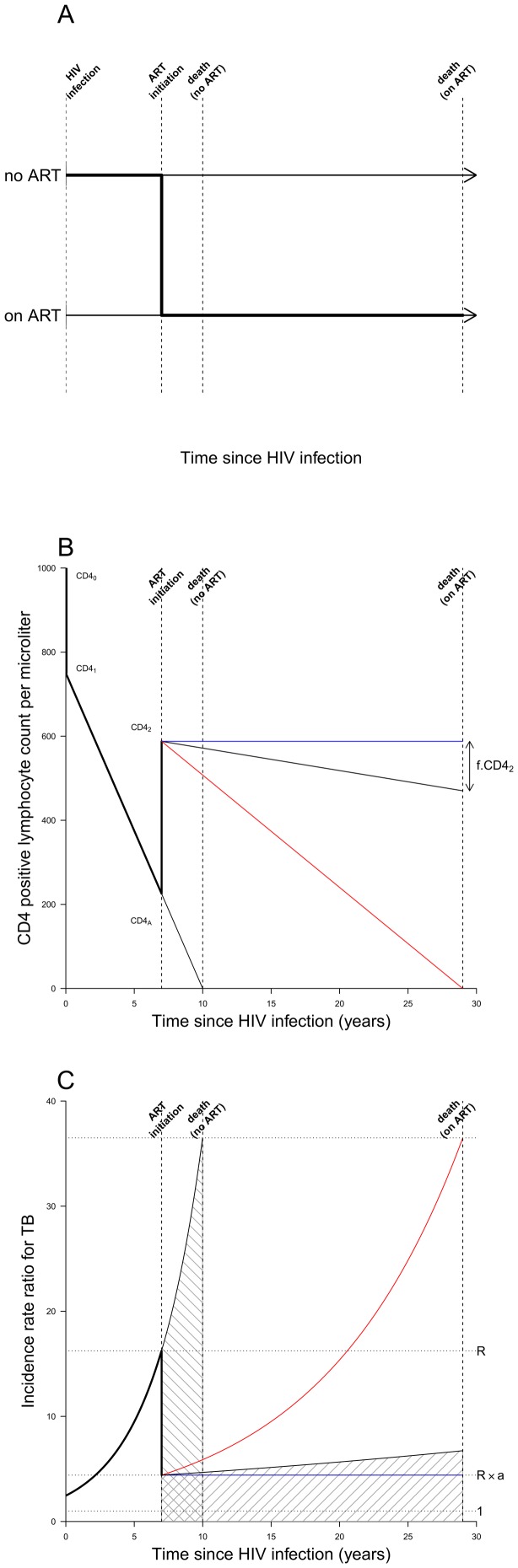
Model structure and assumptions. A. Schematic of modelled progression from HIV infection to death. People living with HIV are modelled using a continuous time-since-infection. Their time-of-death without ART, is drawn from a Weibull distribution, which determines their rate of CD4 cell count decline (see Figure 1B). When CD4 cell count reaches some threshold value CD4_A_ individuals may start ART with probability determined by the coverage at this calendar-time, in which case their time-of-death is postponed to a time dependent on their age and CD4 count and their CD4 count dynamics altered (see Figure 1B). **B. Model of CD4 positive lymphocyte count by time since HIV infection.** CD4 counts (cells per microliter) are assumed to begin at CD4_0_ = 1000, drop by 25% upon infection, and decline linearly to zero at death in the absence of treatment. ART begins at a threshold CD4_A_ and is modelled as instantaneously increasing CD4 cell count to the level achieved after immune reconstitution has plateaued, CD4_2_. To explore the uncertainty in the durability of this recovery due to treatment failure and loss to follow-up, the mean cohort CD4 cell count is assumed to linearly decline resulting in the loss of a fraction *f* of CD4_2_ on death. The *optimistic* (blue line, *f* = 0) corresponds to fully maintained CD4 recovery. The *pessimistic* (red line, *f* = 1) corresponds to the eventual complete loss of CD4 cells. **C.** **The model of the TB incidence rate ratio (IRR) by time since HIV infection.** The IRR for TB (relative those not infected by HIV) is taken to increase exponentially with loss of CD4 count, from an initial value of around 2.5 caused by the initial CD4 drop. The increase in CD4 count associated with starting ART reduces the IRR by a factor *a*, from *R* (just before ART) to a value of 4.4, in line with [10]. The red and blue lines correspond to the *pessimistic* and *optimistic* CD4 trajectories shown in the same colors in Figure 1B. The area with forward-slanting hatching is the lifetime risk of TB on ART; the area with backward-slanting hatching is the lifetime risk of TB for an individual who does not start ART.

We used deterministic partial differential equations to model a population of HIV-infected individuals, as HIV incidence declined exponentially, and coverage of ART increased. Time-since-infection, age and CD4 cell count are tracked through calendar time, with a variable proportion of those reaching a threshold CD4 count beginning ART (Figure 1). Reinfection, reactivation and primary routes to TB disease are included as an average, under the assumptions that the TB force-of-infection is constant and that each route is affected by HIV in the same way. HIV incidence is modelled as an independent function of time, and TB incidence in HIV-uninfected individuals is not modelled explicitly.

The age of HIV infection is modelled by a Weibull distribution with shape parameter *k = *2.3 and scale *s = *25.9 years matching the gender-average of data presented in *Stover et al*. 2010 [16]. Life expectancy without ART is modelled by a Weibull distribution with parameters (*k = *2.3, *s = *13.3 years), from a weighted least-squares fit to the survival data from the *CASCADE* collaboration [17]. The CD4 cell count trend and rate ratio for developing TB in HIV-infected individuals not receiving ART are modelled similarly to *Williams et al.* [15]. Upon infection, CD4 cell count drops by 25% from an initial value of 1000 cells per microliter, followed by a linear decline to zero at death. Individuals’ TB incidence rate increases exponentially with decreasing CD4 cell count with a rate 0.36 per 100 cells per microliter.

ART is assumed to start only when individuals reach a threshold CD4 count (CD4_A_ = 225 cells per microliter in default scenarios; 100, 350 and 500 cells per microliter scenarios in Figures S5 and S6 in Materials S1), and ART scale-up is modelled by an exponential decline through calendar time, *t*, in the proportion of individuals who do not start ART when they pass this threshold, with no one receiving ART at *t = *0 (Figure S4 in Materials S1). Those already having CD4 counts below the threshold at *t = *0 do not receive ART. Our default ART scale-up occurred over 5 years. TB risk by CD4 count is assumed to be the same on and off ART. As our primary focus is the long-term consequences, we assume the two year immune recovery on starting ART reported in [18], [19] is approximated by an instantaneous increase in CD4 count to a level compatible with observations of TB risk on ART after CD4 cell count recovery has occurred, i.e. so that the incidence rate ratio (IRR) for HIV-infected vs. HIV-uninfected is 4.4 [10], corresponding to a CD4 count of 588 cells per microliter (Figure 1B, CD4_2_). The ratio of the TB rate just after ART initiation to the rate, *R*, just before ART initiation is denoted *a*. For an averaged representation of waning protection from TB due to immunologic failure and imperfect adherence, we allow TB incidence to increase exponentially while on ART, varying between our *optimistic* scenario (*f* = 0, *blue* lines in Figure 1), which has perfectly maintained protection on ART, and our *pessimistic* scenario (*f* = 1, red lines in Figure 1). The corresponding mean CD4 count of cohorts after starting ART is shown with matching colors in Figure 1B: *f* represents the fractional CD4 decline from post-ART immune recovery to death.

Life expectancy on ART is influenced by age and CD4 cell count [20], [21]. We model life expectancy as *(60-age)*. *l^p^* where *age* is the current age, and *l* the fraction of life expectancy remaining when starting ART (a measure of CD4 depletion in the model). The parameter *p* controls how strongly late ART initiation reduces life expectancy from the level of HIV-uninfected individuals, and is taken to be 0.5 in the default scenario (see Figure S1 in Materials S1). When *p*<1, there is a stronger influence of a low CD4 count at ART start on life expectancy compared with a high CD4 count; increasing *p* results in shorter life expectancy (see Figure S2 in Materials S1).

Given the above assumptions about CD4 decline by time-since-infection and the effect of ART on TB rates and life expectancy, the partial differential equations describing TB incidence in a cohort with specified age at HIV infection and HIV life expectancy without ART were solved analytically. To compute TB incidence for a population with distributions of HIV life expectancy and age at infection, averages over the Weibull distributions describing these variables’ statistical variability were calculated by Monte Carlo methods to within an accuracy of <1%, using the R statistical package [22] (see Materials S1 for more details). To ensure conclusions were not influenced by secular trends, we started the model from an equilibrium with constant HIV and TB incidences, and a constant birth rate. 50 year TB incidence time-series were generated for scenarios with 0% and 10% per year rates of decline in HIV incidence. Peak and cumulative TB incidence over 50 years were explored as the decline in HIV incidence varied from 0% to 25% per year and the long-term protection on ART varied from perfect to zero efficacy. Sensitivity of these outputs to uncertainties in the durability of protection on ART and life expectancy on ART was considered by varying parameters *f* and *p* from 0 to 1.

## Results

**Figure 2:**
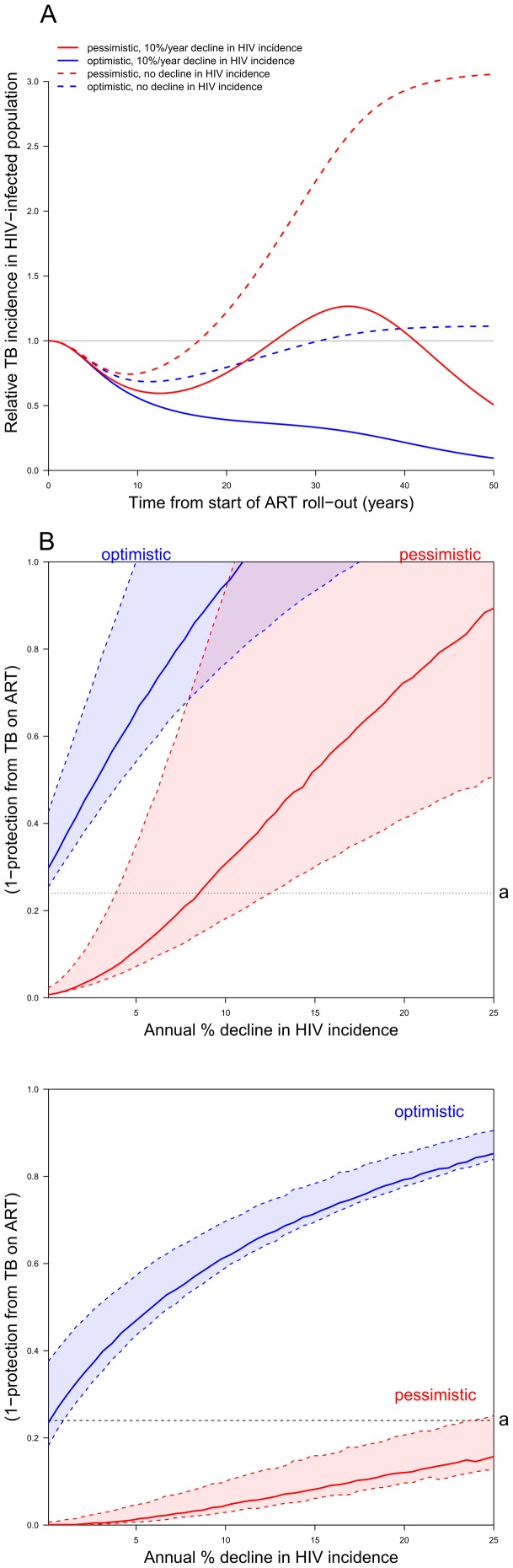
Results from the model. A. TB incidence dynamics. Annual population-level TB incidence among HIV-infected persons relative to the value before ART scale up. ART coverage, with initiation at CD4 count of 225 cells per microliter, is assumed to increase from 0% to 100% over 5-years. Blue and red lines correspond to the pessimistic and optimistic scenarios of Figures 1B & C. Dashed lines assume no drop in HIV incidence; solid lines correspond to an HIV incidence declining at a proportional rate of 10% per year. **B.** **Contours separating regions where cumulative TB incidence over 50 years is increased from regions where it is decreased,** as the level of protection against TB conferred by ART (IRR) and the decline in HIV incidence vary. The solid colored lines correspond to the default dependence of life expectancy on CD4 count at ART initiation (i.e. *p* = 0.5, see main text). Regions below these lines correspond to decreases in cumulative incidence; above the lines to increases. Red and blue lines correspond to the optimistic and pessimistic scenarios as shown in Figures 1B & C and described in the text. The shaded regions bounded by dashed lines indicate the variation of the contours of zero change for cumulative incidence as the dependency of life expectancy on CD4 count at ART initiation is varied (*p* ranging from 0.25 to 1). Contours sweep from the lower dashed line for longer life expectancies to the upper dashed line at shorter life expectancies. The horizontal black dotted line represents the level of protection after CD4 recovery corresponding to Figures 1B & C, i.e. to *a* = 0.24 for those started at CD4_A_ = 225 cells per microliter. **C.** **Contours separating regions where peak TB incidence over 50 years is higher than baseline from regions where it is lower,** as the level of protection against TB conferred by ART (IRR) and the decline in HIV incidence vary. The conditions and interpretation are as for Figure 2B, but with the outcome being *peak* rather than *cumulative* TB incidence over 50 years: the areas below the lines correspond to situations where the peak incidence does not exceed baseline.

Model predictions of TB incidence relative to its value before ART scale up (*t = *0, Figure 2A) show that with ultimately complete ART coverage and hence lower individual TB incidence rates, total TB incidence would initially decrease in all four depicted scenarios (blue for *optimistic* and red for *pessimistic* scenarios in all figures). In the two scenarios where HIV incidence remains constant (dashed lines in Figure 2A), TB incidence tends towards a new equilibrium whose value is only dependent on the total lifetime risk of developing TB (see Figure S3 and associated explanation in Materials S1). Whether this equilibrium is higher or lower than the initial TB incidence is completely determined by comparing the size of the hatched areas in Figure 1C. This captures the competing effects of the extended life-span and reduced hazard of TB whilst on long-term ART. In the two scenarios where HIV incidence is falling at 10% per year (solid lines in Figure 2A) the TB incidence in HIV-infected people must also eventually tend to zero because the size of the HIV-infected population falls to zero. Thus in scenarios that assume a 10% per year decline in HIV incidence (solid lines in Figure 2A) there is competition between the trend towards a higher equilibrium and the reduction in HIV incidence. Under pessimistic assumptions about maintenance of immune status and hence protection from TB on ART (red solid line in Figure 2A), even large rates of decline in HIV incidence may not prevent a higher peak rebound in population TB incidence in the future (seen here after 30 years).

The cumulative incidence of TB over 50 years could remain unchanged by the introduction of ART if the increase in HIV prevalence is balanced by the lower individual TB rates. This balance is quantitatively mapped in Figure 2B: areas below the solid colored lines (blue for the optimistic scenario, red for the pessimistic), where protection is better (lower y-values), or HIV decline faster (higher x-values), correspond to regions where the cumulative TB incidence is reduced over 50 years. Most scenarios correspond to reductions in cumulative TB incidence when assumptions about durability of protection on ART are optimistic, and for earlier CD4 thresholds for ART initiation (see Figure S5 in Materials S1). This conclusion is robust to plausible variations in life expectancy assumptions (shaded regions bounded by dashed lines).

Similar calculations (Figure S2C and Figure S6 in Materials S1) determine when any peak in TB incidence over 50 years is higher or lower than the initial rate. A lower peak incidence is found to be less common than a reduction in cumulative incidence (a smaller area beneath the solid lines), particularly for scenarios with waning protection.

## Discussion

Our model predicted that extensive ART roll-out will initially reduce TB incidence, but that total TB incidence may under certain circumstances increase after approximately 20 years, when reductions in HIV-associated mortality lead to increased lifetime risks of TB. This subsequent peak in TB incidence may exceed TB incidence before ART roll-out in scenarios with waning protection on ART or slowly declining HIV incidence. Cumulative TB incidence over 50 years is predicted to be lower with ART in most scenarios where HIV incidence declined by more than 10% per year. This suggests that in order to decrease TB incidence in the long-term, HIV preventative strategies such as universal ‘test and treat’ may be key to controlling the HIV-associated TB epidemic.

Our modelling complements that of *Williams et al.* [15] in exploring ranges for key quantities. We vary changes in HIV incidence independently from ART coverage, allowing for more or less effective control of HIV; we consider a range of effects of ART on immune status and the greatest number of people starting long-term ART.ighest prevalence of HIV in[4]; we explore waning protection and different life expectancies on ART, reflecting long-term uncertainties at the individual and programmatic level.

We modelled rates of CD4 decline as correlated with mortality (the random variable for CD4 decline rate was a function of that modelling life expectancy). However our scenario explorations allow the durability of TB protection and the reduction in mortality from ART to vary independently. It may be more realistic to consider longer life expectancies linked to better sustained TB protective effect, and vice versa. In this case, parameter combinations with higher values for both *p* and *f* (the regions towards the lower extent of the blue-, and the upper extent of the red-shaded areas in Figures 2 B&C) would be most relevant. Proportional declines in HIV incidence of more than 5-10% per year would then be sufficient to reduce cumulative TB incidence among people living with HIV, but not to avoid a substantial increase in TB incidence after around 20 years. Such an increase need not imply a concomitant increase in mortality if TB monitoring and treatment programs for those on ART are functioning sufficiently well. Measures that further reduce the risk of TB, such as isoniazid preventive therapy (IPT) for those with HIV [23]–[25], may have a role in countering any rebound in incidence. IPT added to ART has been shown to further reduce TB rates by a supplementary factor of around 0.31–0.63 [23], [24], [26] but without a further large increment to life expectancy. The long-term durability of this effect is unknown, but sustained suppression was observed when IPT was given in combination with ART continuously over 3 years [25]. In Figures 2B and C, the effect on a given choice of protection and HIV decline of adding continuous IPT to ART can therefore be assessed by reducing the IRR (y-value) by this factor. This implies a substantial decrease in the range of parameters for which cumulative TB incidence or peak TB incidence would increase. Earlier ART initiation may also avoid this, depending on the relative protection from TB afforded at higher CD4 counts, as well as the effect on HIV-incidence.

This model assumes a fixed annual risk of tuberculosis infection (ARTI) and does not account for potential changes over time, for example reductions due to improved control of TB among HIV-uninfected individuals. Including TB transmission from HIV-infected individuals would tend to exaggerate both the increases and decreases in TB incidence predicted by this model. It is likely, however, that HIV-infected individuals contribute a small proportion of the ARTI due to the lower rates of smear-positivity [27]–[29] and a shorter duration of disease [27], [30], [31]. Once relevant data become available, future modelling may need to consider different characteristics for HIV-related TB in those on ART, such as different rates of smear positivity and detection.

This paper has focused on long-term trends in TB incidence rather than overall health outcomes, which we do not imply would be worsened by ART scale-up. These data will be particularly relevant for understanding potential trends in TB rates in the countries in southern Africa, which have the highest prevalence of HIV in the world, the highest TB incidence rates, and the greatest number of people starting long-term ART. Our results do not imply that ART scale-up would be disadvantageous, but rather that the possibility of future increases in TB incidence may require bolstering of TB control efforts.

In summary, this model described the potential for ART scale-up to be associated with a rebound in long-term population TB incidence rates driven by increased life expectancy in PLHIV. This highlights the importance of sustaining good long-term immunovirological responses to ART, use of adjunctive TB preventive interventions and reducing HIV incidence with, for example, a strategy of universal test and treat that would offer further opportunities for integrated control.

## Supporting Information

Materials S1 Predicting the long-term impact of antiretroviral therapy scale-up on population incidence of tuberculosis.
(PDF)

## References

[1] World Health Organization (2012) Global tuberculosis control: WHO report 2011. Geneva, Switzerland.

[2] Harries AD, Zachariah R, Corbett EL, Lawn SD, Santos-Filho ET, et al (2010) The HIV-associated tuberculosis epidemic--when will we act? The Lancet 375: 1906–19. 10.1016/S0140-6736(10)60409-6 20488516

[3] Lawn SD, Wood R, De Cock KM, Kranzer K, Lewis JJ, et al (2010) Antiretrovirals and isoniazid preventive therapy in the prevention of HIV-associated tuberculosis in settings with limited health-care resources. The Lancet infectious diseases 10: 489–98. 20610331 10.1016/S1473-3099(10)70078-5

[4] Suthar AB, Lawn SD, Del Amo J, Getahun H, Dye C, et al (2012) Antiretroviral therapy for prevention of tuberculosis in adults with HIV: a systematic review and meta-analysis. PLoS medicine 9: e1001270. 22911011 10.1371/journal.pmed.1001270 PMC3404110

[5] Lawn SD, Harries AD, Williams BG, Chaisson RE, Losina E, et al (2011) Antiretroviral therapy and the control of HIV-associated tuberculosis. Will ART do it? The international journal of tuberculosis and lung disease: the official journal of the International Union against Tuberculosis and Lung Disease 15: 571–81. 10.5588/ijtld.10.0483 PMC4067901 21756508

[6] Lawn SD, Myer L, Bekker LG, Wood R (2006) Burden of tuberculosis in an antiretroviral treatment programme in sub-Saharan Africa: impact on treatment outcomes and implications for tuberculosis control. AIDS (London, England) 20: 1605–12. 10.1097/01.aids.0000238406.93249.cd 16868441

[7] Van Rie A, Westreich D, Sanne IM (2011) Tuberculosis in patients receiving antiretroviral treatment: incidence, risk factors, and prevention strategies. Journal of acquired immune deficiency syndromes 56: 349–355. 20926954 10.1097/QAI.0b013e3181f9fb39 PMC3319435

[8] Middelkoop K, Wood R, Bekker LG (2011)The impact of antiretroviral treatment programs on tuberculosis notification rates. The international journal of tuberculosis and lung disease: the official journal of the International Union against Tuberculosis and Lung Disease: 15:1714; author reply 1714–5. 10.5588/ijtld.11.0545 22118185

[9] Zachariah R, Bemelmans M, Akesson A, Gomani P, Phiri K, et al (2011) Reduced tuberculosis case notification associated with scaling up antiretroviral treatment in rural Malawi. The international journal of tuberculosis and lung disease: the official journal of the International Union against Tuberculosis and Lung Disease 15: 933–7. 10.5588/ijtld.10.0666 21682967

[10] Gupta A, Wood R, Kaplan R, Bekker LG, Lawn SD (2012) Tuberculosis Incidence Rates during 8 Years of Follow-Up of an Antiretroviral Treatment Cohort in South Africa: Comparison with Rates in the Community. PLoS ONE 7: e34156. 22479548 10.1371/journal.pone.0034156 PMC3316623

[11] Nglazi MD, Lawn SD, Kaplan R, Kranzer K, Uk M, et al (2011) Changes in Programmatic Outcomes During 7 Years of Scale-up at a Community-Based Antiretroviral Treatment Service in South Africa. Journal of acquired immune deficiency syndromes 56: 1–8. 21084996 10.1097/QAI.0b013e3181ff0bdc PMC3776048

[12] Cornell M, Grimsrud A, Fairall LR, Fox MP, Van Cutsem G, et al (2011) Temporal changes in programme outcomes among adult patients initiating antiretroviral therapy across South Africa, 2002-2007. AIDS (London, England) 24: 2263–2270. 10.1097/QAD.0b013e32833d45c5 PMC2948209 20683318

[13] Cohen MS, Chen YQ, McCauley M, Gamble T, Hosseinipour M, et al (2011) Prevention of HIV-1 infection with early antiretroviral therapy. New England Journal of Medicine 365: 493–505. 21767103 10.1056/NEJMoa1105243 PMC3200068

[14] Granich R, Gilks C, Dye C, Decock K, Williams B (2009) Universal voluntary HIV testing with immediate antiretroviral therapy as a strategy for elimination of HIV transmission: a mathematical model. The Lancet 373: 48–57. 10.1016/S0140-6736(08)61697-9 19038438

[15] Williams BG, Granich R, De Cock KM, Glaziou P, Sharma A, et al (2010) Antiretroviral therapy for tuberculosis control in nine African countries. Proceedings of the National Academy of Sciences of the United States of America 107: 19485–9. 20974976 10.1073/pnas.1005660107 PMC2984151

[16] Stover J, Johnson P, Hallett T, Marston M, Becquet R, et al (2010) The Spectrum projection package: improvements in estimating incidence by age and sex, mother-to-child transmission, HIV progression in children and double orphans. Sexually transmitted infections 86 Suppl 2ii16–21. 21106510 10.1136/sti.2010.044222 PMC3173821

[17] CASCADE (2000) Time from HIV-1 seroconversion to AIDS and death before widespread use of highly-active antiretroviral therapy: a collaborative re-analysis. The Lancet 355: 1131–7. 10791375

[18] Gras L, Kesselring AM, Griffin JT, Van Sighem AI, Fraser C, et al (2007) CD4 cell counts of 800 cells/mm3 or greater after 7 years of highly active antiretroviral therapy are feasible in most patients starting with 350 cells/mm3 or greater. Journal of acquired immune deficiency syndromes 45: 183–92. 17414934 10.1097/QAI.0b013e31804d685b

[19] Nash D, Katyal M, Brinkhof MWG, Keiser O, May M, et al (2008) Long-term immunologic response to antiretroviral therapy in low- income countries: Collaborative analysis of prospective studies. AIDS (London, England) 22: 2291–2302. 10.1097/QAD.0b013e3283121ca9 PMC2794130 18981768

[20] “HIV-CAUSAL Collaboration” (2012) Impact of Antiretroviral Therapy on Tuberculosis Incidence Among HIV-Positive Patients in High-Income Countries. Clinical infectious diseases: an official publication of the Infectious Diseases Society of America: 54. doi:10.1093/cid/cis203 10.1093/cid/cis203 PMC3404691 22460971

[21] Zaba B, Marston M, Crampin AC, Isingo R, Biraro S, et al (2007) Age-specific mortality patterns in HIV-infected individuals: a comparative analysis of African community study data. AIDS (London, England) 21 Suppl 6S87–96. 10.1097/01.aids.0000299415.67646.26 18032944

[22] R Development Core Team (2009) R: A language and environment for statistical computing. ISBN 3-900051-07-0. R Foundation for Statistical Computing Vienna Austria. 2010; http://www.r-project.org

[23] Golub JE, Pronyk P, Mohapi L, Thsabangu N, Moshabela M, et al (2011) Isoniazid preventive therapy, HAART and tuberculosis risk in HIV-infected adults in South Africa: a prospective cohort. AIDS 23: 631–636. 10.1097/QAD.0b013e328327964f PMC3063949 19525621

[24] Golub JE, Saraceni V, Cavalcante SC, Pacheco AG, Moulton LH, et al (2007) The impact of antiretroviral therapy and isoniazid preventive therapy on tuberculosis incidence in HIV-infected patients in Rio de Janeiro, Brazil. AIDS (London, England) 21: 1441–1448. 10.1097/QAD.0b013e328216f441 PMC3063947 17589190

[25] Samandari T, Agizew TB, Nyirenda S, Tedla Z, Sibanda T, et al (2011) 6-month versus 36-month isoniazid preventive treatment for tuberculosis in adults with HIV infection in Botswana: a randomised, double-blind, placebo-controlled trial. The Lancet 377: 1588–98. 10.1016/S0140-6736(11)60204-3 21492926

[26] Rangaka M, Wilkinson R, Wilkinson K, Glynn J, Boulle A, et al.. (2013) Effect of Tuberculin Skin Testing or Interferon-release on the Benefit of Concurrent Isoniazid Preventive Therapy with ART: Subgroup Analysis of a Randomized Controlled Trial. 2013; CROI abstract #189LB.

[27] Corbett EL, Watt CJ, Walker N, Maher D, Williams BG, et al (2003) The growing burden of tuberculosis: global trends and interactions with the HIV epidemic. Archives of internal medicine 163: 1009–21. 12742798 10.1001/archinte.163.9.1009

[28] Espinal M A, Peréz EN, Baéz J, Hénriquez L, Fernández K, et al (2000) Infectiousness of Mycobacterium tuberculosis in HIV-1-infected patients with tuberculosis: a prospective study. The Lancet 355: 275–80. 10.1016/S0140-6736(99)04402-5 10675075

[29] Getahun H, Harrington M, O’Brien R, Nunn P (2007) Diagnosis of smear-negative pulmonary tuberculosis in people with HIV infection or AIDS in resource-constrained settings: informing urgent policy changes. The Lancet 369: 2042–9. 10.1016/S0140-6736(07)60284-0 17574096

[30] Corbett EL, Charalambous S, Moloi VM, Fielding K, Grant AD, et al. Human immunodeficiency virus and the prevalence of undiagnosed tuberculosis in African gold miners. American journal of respiratory and critical care medicine 170: 673–9. 15191919 10.1164/rccm.200405-590OC

[31] Dimairo M, Mativenga S, Dauya E, Mungofa S, Makamure B, et al. (2009) The Fate of Sputum Smear-negative TB Suspects Managed by Routine Clinical Services in Harare, Zimbabwe. CROI 2009 Abstract #778. http://retroconference.org/2009/Abstracts/34638.htm (accessed 9 Dec 2010).

