# Correction: Predicting the Long-Term Impact of Antiretroviral Therapy Scale-Up on Population Incidence of Tuberculosis

**DOI:** 10.1371/annotation/814ac2ee-3cac-41f9-80d4-33f6c80dade0

**Published:** 2013-10-01

**Authors:** Peter J. Dodd, Gwenan M. Knight, Stephen D. Lawn, Elizabeth L. Corbett, Richard G. White

Text was introduced into the second paragraph of the Discussion section. The correct version of the paragraph is available below.

"Our modelling complements that of Williams et al. [15] in exploring ranges for key quantities. We vary changes in HIV incidence independently from ART coverage, allowing for more or less effective control of HIV; we consider a range of effects of ART on immune status [4]; we explore waning protection and different life expectancies on ART, reflecting long-term uncertainties at the individual and programmatic level." 

