# Supplementary material for: Predicting the Long-Term Impact of Antiretroviral Therapy Scale-Up on Population Incidence of Tuberculosis
Source: PLoS One. 2013 Sep 17;8(9):e75466. doi: 10.1371/journal.pone.0075466 (PMC3775764; doi:10.1371/journal.pone.0075466)
Supplement: Materials S1 — Predicting the long-term impact of antiretroviral therapy scale-up on population incidence of tuberculosis. (PDF) [file pone.0075466.s001.pdf]

Predicting the long-term impact of antiretroviral therapy scale-up on population incidence of tuberculosis. Online appendix.

P.J. Dodd*[1,2], G.M. Knight[1,2], S.D. Lawn[2,3], E.L. Corbett[2,4], and R.G. White[1,2]

[1]Centre for Mathematical Modelling of Infectious Diseases, London School of Hygiene and Tropical Medicine, London, WC1E 7HT, UK
[2]TB Centre, London School of Hygiene and Tropical Medicine, London, WC1E 7HT, UK
[3]Desmond Tutu HIV Centre, University of Cape Town, Anzio Road Capetown, South Africa
[4]Malawi-Liverpool-Wellcome, Trust Clinical Research Programme, Blantyre, Malawi

Contents

1 Model of life-expectancy **2**

2 Model of TB risk and CD4 positive cell count decline **2**

3 HIV/ART model **5**
- 3.1 HIV only 5
- 3.2 ART population 6
- 3.3 Combined solution 7
- 3.4 Starting from equilibrium 7

*e-mail: peter.dodd@lshtm.ac.uk

4 TB incidence **9**
4.1 Equilibrium incidence 9
4.2 Specific solution 10

5 Supplementary results **14**

6 Comparison of notation **14**

1 Model of life-expectancy

Mortality on ART has been shown to depend strongly on age and CD4 cell count at initiation [1, 4]. We construct a life-expectancy at ART initiation that follows the general patterns described in [1], while allowing flexible variation. We take life-expectancy on starting ART to be

$$\begin{aligned} LE =& [60 - a]_+ \times (\tau_A/\tau_m)^\gamma \\ =& [60 - a]_+ \times \left(\frac{CD4_A}{CD4_1}\right)^\gamma \end{aligned} \tag{1}$$

where $[X]_+ = \max(X, 0)$, and where $CD4_A$ and $CD4_1$ are points in CD4-count schedule described in Section 2 and Figure 1B in the main text. This means that those who state ART later, for a given age, live less long than those who start early. More life-years are lost to later starts as γ increases. For $\gamma < 1$, differences in CD4 counts for ART initiation have a greater impact on survival at low CD4 counts than at high CD4 counts.

Figure 1 plots life-expectancies for different ages at ART starts for the default $\gamma = 0.5$, and Figures 2a & 2b plot life-expectancies for the two extremes of the range of γ considered ($\gamma = 0.25$ and $\gamma = 1.00$, respectively).

2 Model of TB risk and CD4 positive cell count decline

We assume HIV-uninfected individuals have a CD4 cell count of 1000 cells/μL and that, as in [3], this declines immediately by 25% upon HIV infection. Their CD4 cell count is then assumed to decline linearly to death at τ_m.

Upon starting ART, an individual's CD4 cell count is assumed to be boosted by an amount Δ. In the main paper Δ is chosen in conjunction with the risk model of Section 4 to produce the required rate ratio for TB on ART. Given our interest in the long-term, we caricature an increase that in

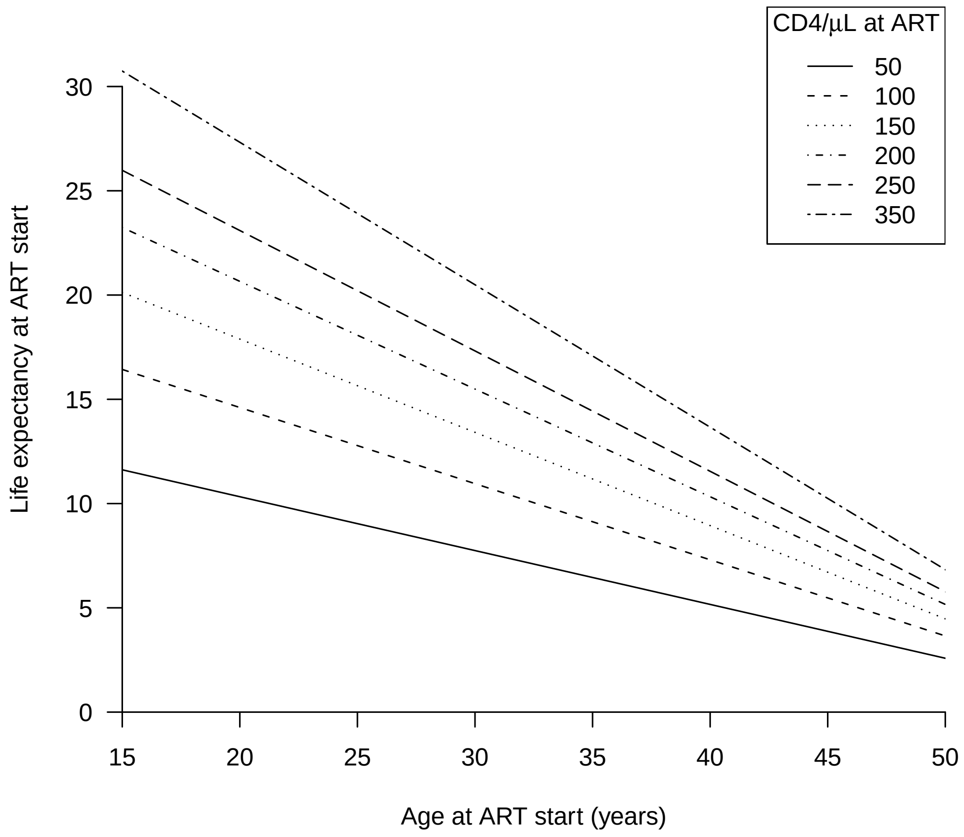

Figure 1: $\gamma = 0.5$.The default life-expectancy on starting ART, as a function of age and CD4 cell count.

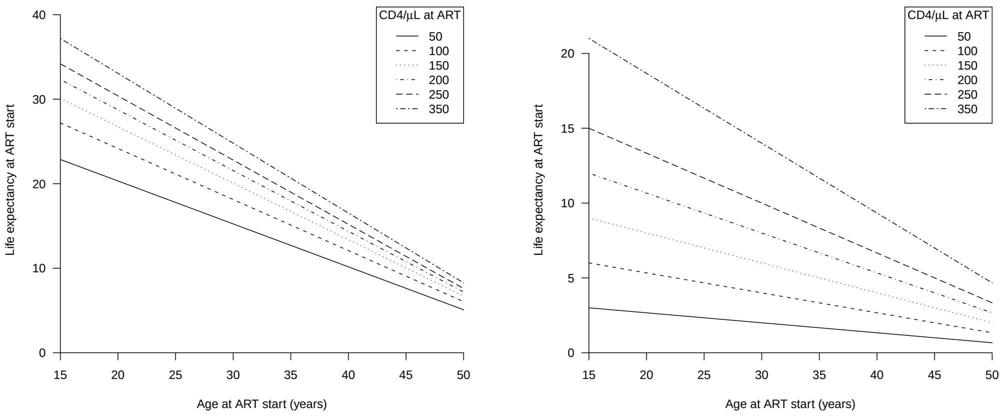

(a) $\gamma = 0.25$

(b) $\gamma = 1$

Figure 2: Alternative scenarios for life-expectancy starting ART.

reality occurs over a few years [2] after starting ART as instantaneous. The CD4 level achieved and the associated protection from TB should therefore be characteristic of a patient on ART once this initial rise has taken place.

A linear decline over the individual's lifetime on ART is assumed to result in the loss of a fraction f of this new CD4 count at time of death τ'_m, due to immunologic failure, imperfect adherence, or other reasons. $f = 1$ corresponds to our 'pessimistic' scenario, wherein an individual's CD4 count declines to zero, although at a slower rate than untreated individuals. $f = 0$ corresponds to our 'optimistic' scenario, wherein the level of CD4 reconstitution and protection from TB are maintained throughout an individual's life, until death at τ'_m.

This implies:

$$CD4_A = CD4_1 \frac{\tau_m - \tau_A}{\tau_m} \tag{2}$$

$$CD4_1 = \frac{3}{4} CD4_0 \tag{3}$$

$$CD4_2 = CD4_A + \Delta \tag{4}$$

$$CD4(\tau) = \begin{cases} CD4_1 \left[\frac{\tau_m - \tau}{\tau_m}\right]_+ & \text{if } 0 < \tau < \tau_A \text{ or off-ART} \\ (1-f).CD4_2 + f.CD4_2 \frac{\tau'_m - \tau}{\tau'_m - \tau_A} & \text{if on ART \& } \tau \geq \tau_A \end{cases} \tag{5}$$

3 HIV/ART model

Figure 1A in the main text summarizes the logic of the model. Individuals are either in class I - infected with HIV, not on ART; or class A - infected with HIV, on ART. New individuals enter the model on the left at a rate that may vary with calendar time, and propagate rightwards. Those who are not started on ART die at point τ_m, whereas those who are started on ART transit to class A at point τ_A and die at a later point τ_m'. The mathematical details are described in the sequel.

3.1 HIV only

Let $I(t, a, \tau; \tau_m)$ be the number of individuals at time t that are aged a, where infected with HIV time τ ago, are not on ART and who will die a time τ_m after their initial infection in the absence of treatment. This quantity satisfies the partial differential equation

$$\left(\frac{\partial}{\partial t} + \frac{\partial}{\partial \tau} + \frac{\partial}{\partial a}\right) I(t, a, \tau; \tau_m) = -\mu(\tau) I(t, a, \tau; \tau_m), \tag{6}$$

where the mortality is defined in terms of a Dirac delta function enforcing death τ_m years after infection:

$$\mu(\tau) = \delta(\tau - \tau_m), \tag{7}$$

and with boundary condition

$$I(t, a, 0; \tau_m) = i(t)\phi(a)P(\tau_m). \tag{8}$$

Here, $i(t)$ is the HIV incidence at calendar time t, $\phi(a)$ represents the distribution of ages at HIV infection, and $P(\tau_m)$ the distribution of life-expectancy at HIV-infection in the absence of treatment. The distributions P and ϕ are modelled as Weibull distributions (as described in the main text), and averages over these distributions performed as described later in this document.

Defining

$$\theta(x) = \begin{cases} 1 & \text{if } x > 0 \\ \frac{1}{2} & \text{if } x = 0 \\ 0 & \text{otherwise} \end{cases} \tag{9}$$

Equations 6 & 8 can be solved by the method of characteristics to give

$$I(t,a,\tau;\tau_m) = \tilde{I}(t,a,\tau;\tau_m)\theta(\tau_m - \tau) \tag{10}$$

$$\tilde{I}(t,a,\tau;\tau_m) = \tilde{I}(t-\tau, a-\tau, 0;\tau_m)\theta(t-\tau) + \tilde{I}(0, a-t, \tau-t;\tau_m)\theta(\tau_m-\tau) \tag{11}$$

with

$$\tilde{I}(t,a,0;\tau_m) = i(t)\phi(a)P(\tau_m) \tag{12}$$

and $\tilde{I}(0,a,\tau;\tau_m)$ determined by the initial condition.

3.2 ART population

We imagine that a proportion $V(t)$ at time t of those reaching a threshold time-since-infection $\tau = \tau_A(\tau_m)$ commence ART. The dependence of τ_A on τ_m allows this threshold to be specified in terms of a CD4 cell count threshold, given a model that determines CD4 cell count in terms of τ and τ_m, like Equation 5. We assume that a new time of death τ'_m is determined by the age, a, time of ART commencement, τ_A, and original time of death τ_m. Given our dependence of τ_A on τ_m, we can treat the new time of death as a function $\tau'_m(a,\tau_m)$. Analogously to Equation 6, $A(t,a,\tau;\tau_m)$, the number of individuals on ART at time t with age a, who were HIV-infected a time τ ago, and with time of death without ART τ_m, satisfies

$$\left(\frac{\partial}{\partial t} + \frac{\partial}{\partial \tau} + \frac{\partial}{\partial a}\right) A(t,a,\tau;\tau_m) = -\mu_A(\tau)A(t,a,\tau;\tau_m), \tag{13}$$

with

$$\mu_A(\tau) = \delta(\tau - \tau'_m) \tag{14}$$

and

$$A(t,a,\tau_A;\tau_m) = V(t)I(t,a,\tau_A;\tau_m) \tag{15}$$

with the right hand side of Equation 15 understood to be evaluated with τ just less than τ_A and imposing $A(t,a,\tau;\tau_m) = 0$ for $\tau < \tau_A$.

3.3 Combined solution

If at $t = 0$ no-one is on ART, the overall solution for A and I is specified by

$$I(t,a,\tau;\tau_m) = \begin{cases} \tilde{I}(t,a,\tau;\tau_m)\theta(\tau_m - \tau) & \text{if } \tau \leq \tau_A \\ (1 - V(t-\tau+\tau_A))\tilde{I}(t,a,\tau;\tau_m)\theta(\tau_m - \tau) & \text{if } \tau > \tau_A \end{cases} \tag{16}$$

$$\begin{aligned} \tilde{I}(t,a,\tau;\tau_m) =& \tilde{I}(t-\tau, a-\tau, 0;\tau_m)\theta(t-\tau) \\ &+ \tilde{I}(0, a-t, \tau-t;\tau_m)\theta(\tau_m - \tau) \qquad (17) \\ A(t,a,\tau;\tau_m) =& A(t-\tau+\tau_A, a-\tau+\tau_A, \tau_A;\tau_m) \\ &\times \theta(\tau'_m(a-\tau+\tau_A,\tau_m) - \tau) \qquad (18) \end{aligned}$$

and

$$\begin{aligned} &A(t-\tau+\tau_A, a-\tau+\tau_A, \tau_A;\tau_m) \\ &\quad = V(t-\tau+\tau_A)\tilde{I}(t-\tau+\tau_A, a-\tau+\tau_A, \tau_A;\tau_m) \qquad (19) \\ &= V(t-\tau+\tau_A)\times \\ &[\tilde{I}(t-\tau, a-\tau, 0;\tau_m)\theta(t-\tau) + \tilde{I}(0, a-t, \tau-t;\tau_m)\theta(\tau-t)] \qquad (20) \end{aligned}$$

with the boundary condition for $\tilde{I}$ specified by Equation 8.

3.4 Starting from equilibrium

If the system begins at equilibrium at $t = 0$ with HIV incidence equal to i_0, a constant birth-rate, and no-one on ART, and thereafter the HIV incidence follows Equation 8, then this solution becomes

$$\begin{aligned} \tilde{I}(t,a,\tau;\tau_m) =& P(\tau_m)\phi(a-\tau)[i(t-\tau)\theta(t-\tau) + i_0\theta(\tau-t)] \qquad (21) \\ I(t,a,\tau;\tau_m) =& \tilde{I}(t,a,\tau;\tau_m)\theta(\tau_m-\tau)\theta(\tau_A-\tau) \\ &+ (1 - V(t-\tau+\tau_A))\tilde{I}(t,a,\tau;\tau_m)\theta(\tau_m-\tau)\theta(\tau-\tau_A) \qquad (22) \\ A(t,a,\tau;\tau_m) =& V(t-\tau+\tau_A)P(\tau_m)\phi(a-\tau)\theta(\tau'_m(a-\tau+\tau_A,\tau_m) - \tau) \\ &\times [i(t-\tau)\theta(t-\tau) + i_0\theta(\tau-t)]\theta(\tau-\tau_A) \qquad (23) \end{aligned}$$

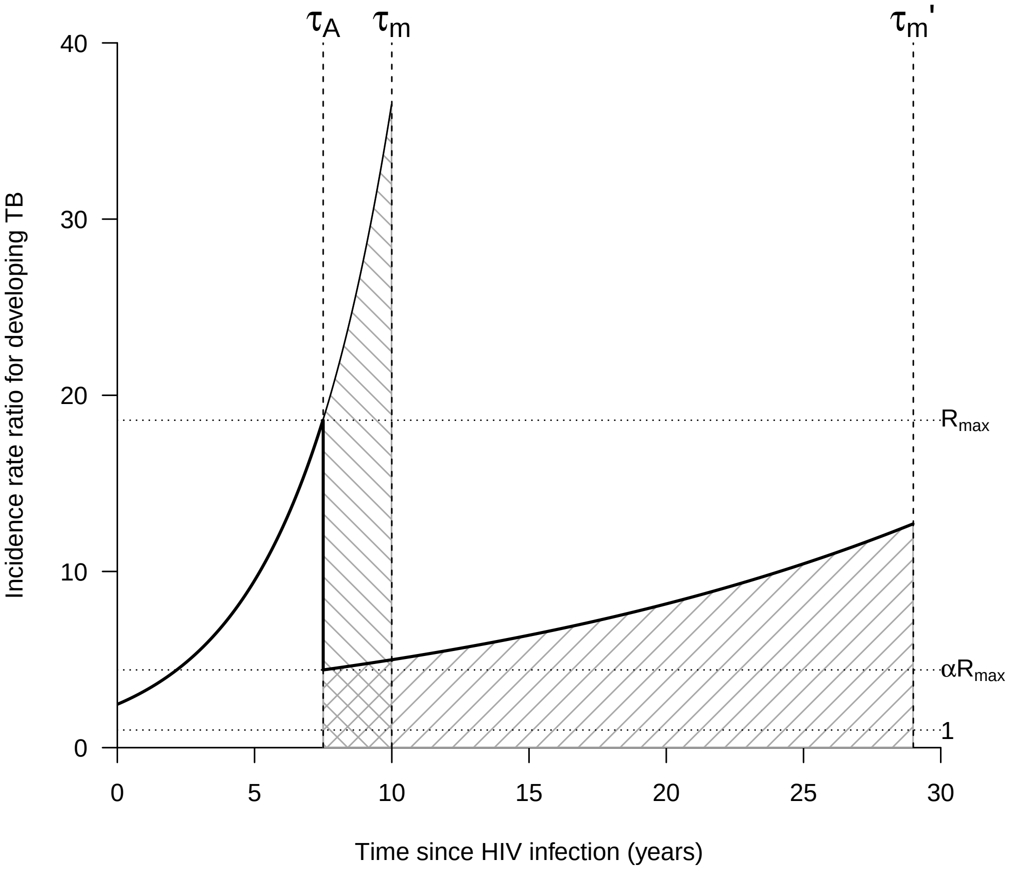

Figure 3: Risk Model: the bold line shows the evolution of the incidence risk ratio of developing TB for an individual who would have lived to time τ_m, but starts ART at τ_A and has their life extended to τ'_m. Significance of the hatched areas is described in Section 4.1.

4 TB incidence

We will take the hazard ratio for developing incident TB to be different according to whether on or off ART, and functions of τ and τ_m (τ'_m for those on ART), denoted: $h_H(\tau, \tau_m)$ and $h_A(\tau, \tau'_m)$ respectively. Let H denote the respective cumulative hazards.

The total TB incidence in those infected with HIV, $\dot{D}(t)$ is given by

$$\dot{D}(t) = \left(\dot{D}_H(t) + \dot{D}_A(t)\right) \tag{24}$$

$$\dot{D}_H(t) = \int_0^\infty d\tau_m P(\tau_m) d_H(t, \tau_m) \tag{25}$$

$$\dot{D}_A(t) = \int_0^\infty d\tau_m P(\tau_m) d_A(t, \tau_m) \tag{26}$$

$$d_H(t, \tau_m) = \int_0^\infty da \int_0^a d\tau I(t, a, \tau; \tau_m) h_H(\tau, \tau_m) \tag{27}$$

$$d_A(t, \tau_m) = \int_0^\infty da \int_0^a d\tau A(t, a, \tau; \tau_m) h_A(\tau, \tau'_m(a - \tau + \tau_A, \tau_m)) \tag{28}$$

4.1 Equilibrium incidence

We calculate the new equilibrium reached after the introduction of ART, when HIV incidence is constant. Writing $i(0) = i_0$, and $\bar{V} = (1 - V)$ for the proportion not starting ART, and using Equations 21 & 22, d_H becomes

$$d_H(t, \tau_m)/i_0 = \int_0^\infty d\tau \theta(\tau_m - \tau)[\theta(\tau_A - \tau) + \bar{V}(t - \tau + \tau_A)\theta(\tau - \tau_A)] h_H(\tau, \tau_m)$$

$$= \int_0^{\tau_A} d\tau h_H(\tau, \tau_m) + \int_{\tau_A}^{\tau_m} d\tau \bar{V}(t - \tau + \tau_A) h_H(\tau, \tau_m)$$

$$= H_H(\tau_A, \tau_m) + \int_{\tau_A}^{\tau_m} d\tau \bar{V}(t - \tau + \tau_A) h_H(\tau, \tau_m) \tag{29}$$

since $\int da.\phi(a) = 1$.

Similarly, using Equation 23, $d_A(t, \tau_m)$ becomes

$$
\begin{aligned}
d_A(t,\tau_m)/i_0 &= \int da d\tau A(t,a,\tau;\tau_m) h_A(\tau,\tau'_m(a-\tau+\tau_A,\tau_m)) \\
&= \int da \int d\tau h_A(\tau,\tau'_m(a-\tau+\tau_A,\tau_m)) \\
&\times V(t-\tau+\tau_A)\phi(a-\tau)\theta(\tau-\tau_A) \\
&= \int_0^\infty dx\phi(x) \int_{\tau_A}^{\tau'_m \wedge (t+\tau_A)} d\tau h_A(\tau,\tau'_m(x+\tau_A,\tau_m)) V(t-\tau+\tau_A) \qquad (30)
\end{aligned}
$$

where we have used the substitution $x = a - \tau$, and used the shorthands $\tau'_m = \tau'_m(x+\tau_A,\tau_m)$ and $a \wedge b = \min(a,b)$.

After a long time ($\sim$ 20 years, see Figure 1(c) in the main text), if $V(t)$ tends to the value V, the sum of d_A and d_H from Equations 29 & 30 becomes (up to an overall factor of i_0):

$$
\begin{aligned}
& H_H(\tau_A,\tau_m) + (1-V).(H_H(\tau_m,\tau_m) - H_H(\tau_A,\tau_m)) \\
& + V. \int_0^\infty dx\phi(x) \left[H_A(\tau,\tau'_m)\right]_{\tau_A}^{\tau'_m} \qquad (31)
\end{aligned}
$$

with $\tau'_m = \tau'_m(x+\tau_A,\tau_m)$. This is easily interpreted in terms of areas under portions of the hazard curves in Figure 3: the first term is the area under the curve up to $\tau = \tau_A$; the second term is $(1-V)$ times the area under the curve from τ_A to τ_m; the last term is V times an age-average of the area under the ART hazard curve from τ_A to τ'_m. It is thus clear that the equilibrium TB incidence will be higher after ART roll-out when the population mean of the forward-hatched area exceeds the mean of the backward-hatched area.

4.2 Specific solution

Following [3], we take TB risk to depend exponentially on the decline in CD4 cell counts:

$$
h(\tau) = \exp\left[\tilde{\rho}(CD4_0 - CD4(\tau))\right] \qquad (32)
$$

with $\tilde{\rho} = 0.36$ per 100 CD4 cells/μL lost.

The TB incidences in the model depend directly only on the model of hazard ratios through time (i.e. Figure 3). We have arrived at these via a model of CD4-decline and the an assumption that CD4 cell count exponentially influences the rate of TB in the same way for HIV-infected individuals regardless of whether they are on ART. For the model of CD4 cell count decline

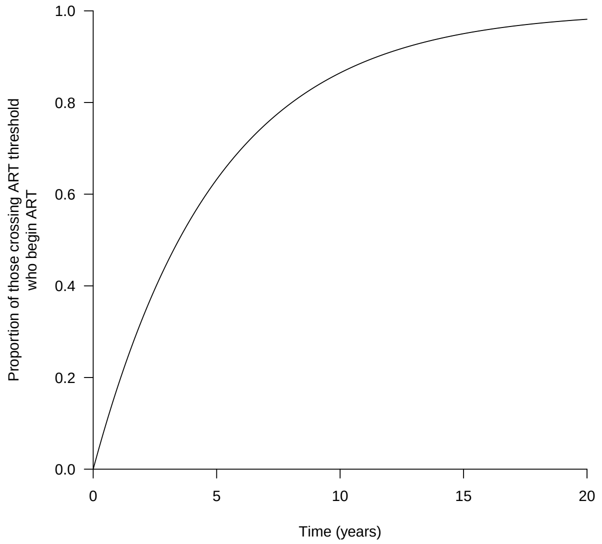

Figure 4: The coverage function for ART, $V(t)$, with $\epsilon = 5$ years.

specified by Equation 5, our hazards are exponential in time-since-infection, τ:

$$h_H(\tau, \tau_m) = h_0. \exp(\rho\tau/\tau_m) \tag{33}$$

$$h_A(\tau, \tau'_m) = \alpha.h_0. \exp(\rho'\tau) \tag{34}$$

We will also take $V(t)$ to be:

$$V(t) = V.\theta(t)(1 - e^{-t/\epsilon}) \tag{35}$$

illustrated for $\epsilon = 5$ years in Figure 4, and HIV incidence to be declining at a rate r for $t > 0$:

$$i(t) = i_0.e^{-rt} \tag{36}$$

Then, from Equation 27

$$d_H(t, \tau_m)/i_0 = \int_0^{\tau_m} d\tau[1 - V.\theta(\tau - \tau_A)\theta(t - \tau + \tau_A)(1 - e^{-(t-\tau+\tau_A)/\epsilon})]$$
$$\times [e^{-r(t-\tau)}\theta(t - \tau) + \theta(\tau - t)]h_H(\tau, \tau_m)$$

$$= h_0 \int_0^{\tau_m \wedge t} d\tau e^{\rho\tau/\tau_m} e^{-r(t-\tau)} + h_0 \int_t^{\tau_m \vee t} d\tau e^{\rho\tau/\tau_m}$$
$$+ h_0 V \int_{\tau_A}^{\tau_m \wedge t} d\tau e^{\rho\tau/\tau_m} e^{-r(t-\tau)} e^{-(t-\tau+\tau_A)/\epsilon} + h_0.V \int_{\tau_A \vee t}^{\tau_m \wedge (\tau_A + t)} d\tau e^{\rho\tau/\tau_m} e^{-(t-\tau+\tau_A)/\epsilon}$$
$$- h_0 V \int_{\tau_A}^{\tau_m \wedge t} d\tau e^{\rho\tau/\tau_m} e^{-r(t-\tau)} - h_0.V \int_{\tau_A \vee t}^{\tau_m \wedge (\tau_A + t)} d\tau e^{\rho\tau/\tau_m}$$

$$= h_0 e^{-rt} \left[\frac{e^{(\rho/\tau_m + r)\tau}}{(\rho/\tau_m + r)} \right]_0^{\tau_m \wedge t} + h_0 \left[\frac{e^{(\rho/\tau_m)\tau}}{(\rho/\tau_m)} \right]_t^{\tau_m \vee t}$$
$$+ h_0 V e^{-(t+\tau_A)/\epsilon - rt} \left[\frac{e^{(\rho/\tau_m + 1/\epsilon + r)\tau}}{(\rho/\tau_m + 1/\epsilon + r)} \right]_{\tau_A}^{\tau_m \wedge t}$$
$$+ h_0.V e^{-(t+\tau_A)/\epsilon} \left[\frac{e^{(\rho/\tau_m + 1/\epsilon)\tau}}{(\rho/\tau_m + 1/\epsilon)} \right]_{\tau_A \vee t}^{\tau_m \wedge (\tau_A + t)}$$
$$- h_0 V e^{-rt} \left[\frac{e^{(\rho/\tau_m + r)\tau}}{(\rho/\tau_m + r)} \right]_{\tau_A}^{\tau_m \wedge t} - h_0.V \left[\frac{e^{(\rho/\tau_m)\tau}}{(\rho/\tau_m)} \right]_{\tau_A \vee t}^{\tau_m \wedge (\tau_A + t)} \quad (37)$$

where it is understood that the integrals and square brackets evaluate to zero when the top limit is smaller than the bottom, i.e.

$$[X(t)]_a^b = \begin{cases} X(b) - X(a) & \text{if } a < b \\ 0 & \text{otherwise} \end{cases} \quad (38)$$

and where $a \vee b = \max(a, b)$.

The contribution to TB incidence from those on ART is similarly given by

$$d_A(t, \tau_m) = \int_0^\infty dx.\phi(x) q(x) \quad (39)$$

where

$$
\begin{aligned}
q(x) =& \\
&\int d\tau V(t-\tau+\tau_A)\theta(\tau-\tau_A)\theta(\tau'_m-\tau)h_A(\tau,\tau'_m)[e^{-r(t-\tau)}\theta(t-\tau)+\theta(\tau-t)] \\
&= V\alpha h_0 \int_{\tau_A}^{z} d\tau(1-e^{-(t-\tau+\tau_A)/\epsilon})e^{-r(t-\tau)}e^{\rho'.\tau} \\
&\quad + V\alpha h_0 \int_{z}^{y} d\tau(1-e^{-(t-\tau+\tau_A)/\epsilon})e^{\rho'.\tau} && (40)\\
&= V\alpha h_0 e^{-rt}\left[\frac{e^{(\rho'+r)\tau}}{(\rho'+r)}\right]_{\tau_A}^{z} + V\alpha h_0\left[\frac{e^{\rho'\tau}}{\rho'}\right]_{z}^{y} \\
&\quad - V\alpha h_0 e^{-(t+\tau_A)/\epsilon - rt}\left[\frac{e^{(\rho'+1/\epsilon+r)\tau}}{(\rho'+1/\epsilon+r)}\right]_{\tau_A}^{z} \\
&\quad - V\alpha h_0 e^{-(t+\tau_A)/\epsilon}\left[\frac{e^{(\rho'+1/\epsilon)\tau}}{(\rho'+1/\epsilon)}\right]_{z}^{y} && (41)
\end{aligned}
$$

with $\tau'_m = \tau'_m(x+\tau_A, \tau_m)$ and $y = \tau'_m \wedge t$ and

$$
z = \begin{cases} \tau_A & \text{if } t < \tau_A \\ t & \text{if } \tau_A < t < y \\ y & \text{if } y < t \end{cases} \tag{42}
$$

or $z = (t \vee \tau_A) \wedge y$.

With the model of CD4 cell count decline described in Section 2, we have

$$
\begin{aligned}
h_A(\tau,\tau'_m) =& \exp\left(\tilde{\rho}CD4_0 - \tilde{\rho}(1-f)CD4_2 - f.\tilde{\rho}CD4_2\frac{\tau'_m-\tau}{\tau'_m-\tau_A}\right) \\
=& h_0\exp\left(\tilde{\rho}CD4_1 - \tilde{\rho}(1-f)CD4_2 + f.\tilde{\rho}CD4_2\frac{\tau'_m-\tau}{\tau'_m-\tau_A}\right) && (43)
\end{aligned}
$$

where

$$
h_0 = \exp[\tilde{\rho}(CD4_0 - CD4_1)] \tag{44}
$$

Comparing with Equation 34, we have

$$
\begin{aligned}
\alpha =& \exp\left[\tilde{\rho}CD4_1 - \tilde{\rho}(1-f)CD4_2 - \tilde{\rho}f.CD4_2\frac{\tau'_m}{\tau'_m-\tau_A}\right] && (45)\\
\rho' =& f\frac{\tilde{\rho}CD4_2}{\tau'_m-\tau_A} && (46)
\end{aligned}
$$

The unevaluated integrals in Equations 25, 26 & 39 can be calculated in a Monte Carlo fashion by sampling τ_m and x from the distributions P and ϕ respectively and taking the mean.

5 Supplementary results

In Figure 5 we display contour plots identical to Figure 1(d) in the main paper, but including different ART initiation thresholds. The IRR, α, marked with the horizontal dashed line corresponds to a protection from ART that brings the TB rate just after ART initiation down to 4.4 times the general population. The uncertainty envelopes arising from varying life-expectancy become broader at low CD4 count since a fixed range of values for γ (see Section 1) results in a larger percentage variation in life-expectancy for lower ART initiation CD4 thresholds, i.e. the model of life-expectancy is more sensitive to the parameter γ for late ART starts.

Figure 6 we display contour plots identical to Figure 1(d) in the main paper, but mapping the regions where any subsequent peak in TB incidence among people living with HIV is higher than the initial level.

6 Comparison of notation

In the main text γ is denoted p, α is denoted a, and l is used to represent (τ_A/τ_m).

References

[1] Edward J Mills, Celestin Bakanda, Josephine Birungi, Keith Chan, Nathan Ford, Curtis L Cooper, Jean B Nachega, Mark Dybul, and Robert S Hogg. Original Research Life Expectancy of Persons Receiving Combination Antiretroviral Therapy in Low-Income Countries : A Cohort Analysis From Uganda. *Annals of internal medicine*, 155:209–216, 2011.

[2] Denis Nash, Monica Katyal, Martin W G Brinkhof, Olivia Keiser, Margaret May, Francois Dabis, Robin Wood, Eduardo Sprinz, and Mauro Schechter. Long-term immunologic response to antiretroviral therapy in low- income countries: Collaborative analysis of prospective studies. *AIDS*, 22(17):2291–2302, 2009.

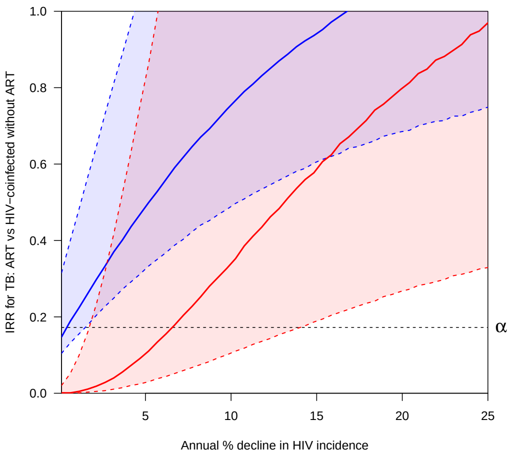

(a) 100 CD4$^+$cells/μL

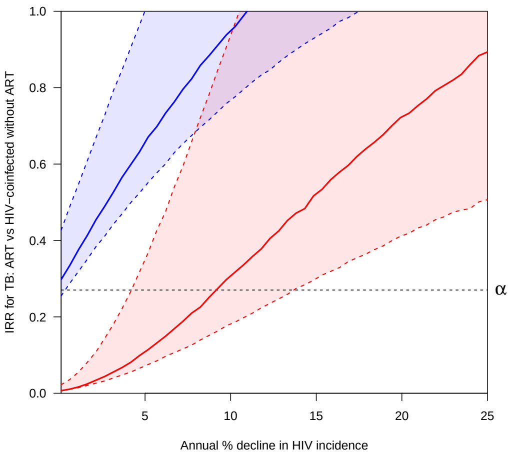

(b) 225 CD4$^+$cells/μL

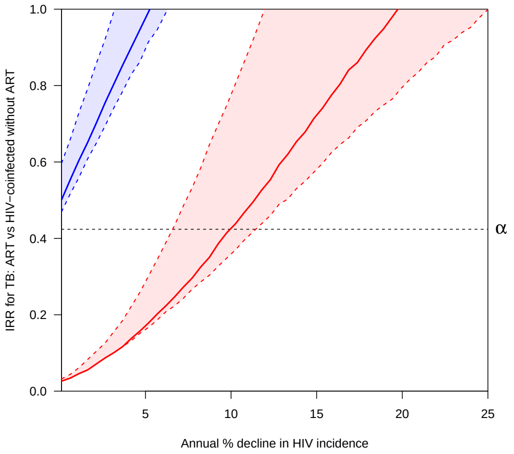

(c) 350 CD4$^+$cells/μL

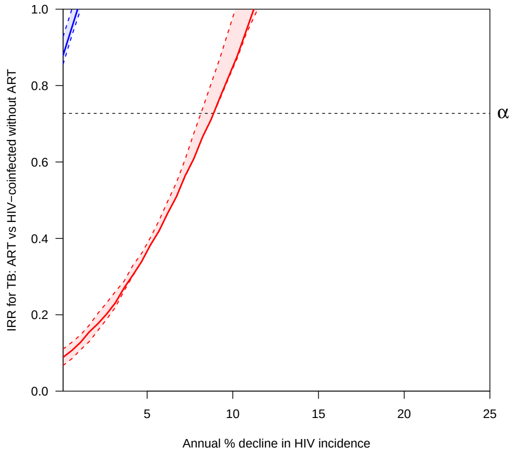

(d) 500 CD4$^+$cells/μL

Figure 5: Contours of regions where the cumulative TB incidence over 50 years is equal to the scenario without ART for different ART initiation CD4 thresholds. Other parameters and interpretation as for Figure 1(d) in the main paper. Below and right of the contours, cumulative incidence is lower than the initial level; above and left of the contours, the peak is higher. See Section 5 for discussion of α.

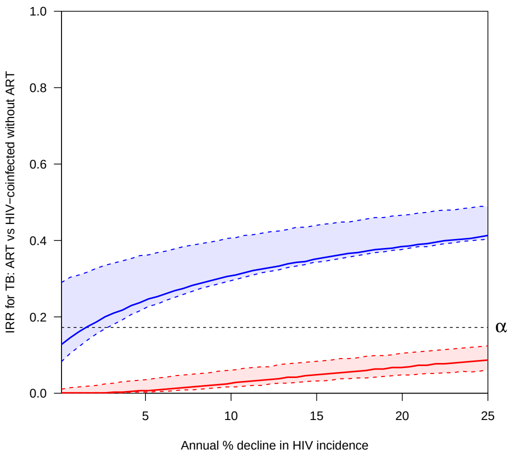

(a) 100 CD4$^+$cells/μL

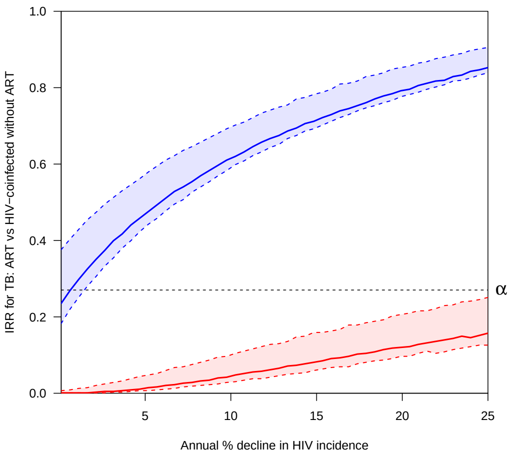

(b) 225 CD4$^+$cells/μL

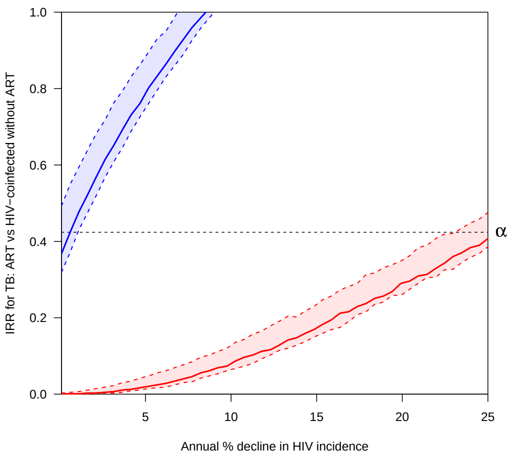

(c) 350 CD4$^+$cells/μL

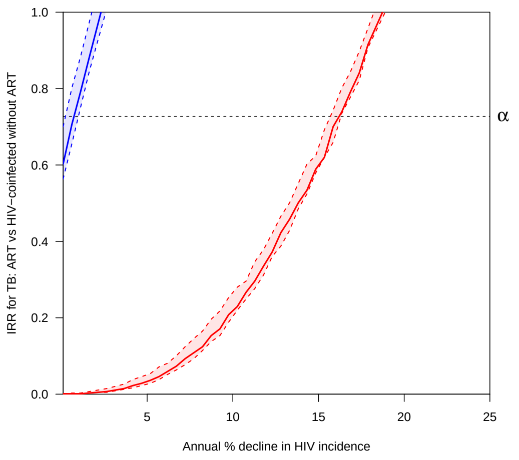

(d) 500 CD4$^+$cells/μL

Figure 6: Contours of regions where the peak in subsequent TB incidence is equal to the initial level for different ART initiation CD4 thresholds. Other parameters and interpretation as for Figure 1(d) in the main paper. Below and right of the contours, any subsequent peak is lower than the initial level; above and left of the contours, the peak is higher. See Section 5 for discussion of α.

[3] Brian G Williams, Reuben Granich, Kevin M De Cock, Philippe Glaziou, Abhishek Sharma, and Christopher Dye. Antiretroviral therapy for tuberculosis control in nine African countries. *Proceedings of the National Academy of Sciences of the United States of America*, 107(45):19485–9, November 2010.

[4] Basia Zaba, Milly Marston, Amelia C Crampin, Raphael Isingo, Sam Biraro, Till Bärnighausen, Ben Lopman, Tom Lutalo, Judith R Glynn, and Jim Todd. Age-specific mortality patterns in HIV-infected individuals: a comparative analysis of African community study data. *AIDS (London, England)*, 21 Suppl 6:S87–96, November 2007.
